# NUC-7738 regulates β-catenin signalling resulting in reduced proliferation and self-renewal of AML cells

**DOI:** 10.1371/journal.pone.0278209

**Published:** 2022-12-15

**Authors:** Akbar Muhammed Shahid, In Hwa Um, Mustafa Elshani, Ying Zhang, David James Harrison

**Affiliations:** 1 School of Medicine, University of St Andrews, St Andrews, United Kingdom; 2 NuCana plc, Edinburgh, United Kingdom; Istanbul University-Cerrahpaşa, Cerrahpaşa Faculty of Medicine, TURKEY

## Abstract

Acute myeloid leukemia (AML) stem cells are required for the initiation and maintenance of the disease. Activation of the Wnt/β-catenin pathway is required for the survival and development of AML leukaemia stem cells (LSCs) and therefore, targeting β-catenin is a potential therapeutic strategy. NUC-7738, a phosphoramidate transformation of 3’-deoxyadenosine (3’-dA) monophosphate, is specifically designed to generate the active anti-cancer metabolite 3’-deoxyadenosine triphosphate (3’-dATP) intracellularly, bypassing key limitations of breakdown, transport, and activation. NUC-7738 is currently in a Phase I/II clinical study for the treatment of patients with advanced solid tumors. Protein expression and immunophenotypic profiling revealed that NUC-7738 caused apoptosis in AML cell lines through reducing PI3K-p110α, phosphorylated Akt (Ser473) and phosphorylated GSK3β (Ser9) resulting in reduced β-catenin, c-Myc and CD44 expression. NUC-7738 reduced β-catenin nuclear expression in AML cells. NUC-7738 also decreased the percentage of CD34^+^ CD38^-^ CD123^+^ (LSC-like cells) from 81% to 47% and reduced the total number and size of leukemic colonies. These results indicate that therapeutic targeting of the PI3K/Akt/GSK3β axis can inhibit β-catenin signalling, resulting in reduced clonogenicity and eventual apoptosis of AML cells.

## Introduction

Acute myeloid leukemia (AML) is a heterogeneous clonal hematopoietic disease characterized by immature myeloid cell proliferation and bone marrow failure [1]. The disease is understood to arise from a series of recurrent genetic alterations within hematopoietic stem and progenitor cells (HSPCs), resulting in the generation of leukemic stem cells (LSCs) [2]. These LSCs survive conventional therapies resulting in disease progression and relapse [3–7]. Thus, extensive investigation has been made towards understanding LSC biology and the signalling mechanisms that control their development and survival [8–10].

One such key pathway is the canonical Wnt/β-catenin pathway [11, 12]. When inactivated in the absence of Wnt ligands, β-catenin stability is governed by the ‘destruction complex’ composed of the scaffold protein Axin, adenomatous polyposis coli (APC), glycogen synthase kinase 3β (GSK3β) and casein kinase 1α (CK1α). When bound to this complex, GSK3β phosphorylates β-catenin, resulting in its ubiquitination by β-TrCP and subsequent proteasomal degradation. The lack of nuclear β-catenin allows for the recruitment of histone deacetylases (HDACs) by the inhibitory complex TCF/LEF and transducing-like enhancer protein (TLE/Groucho), resulting in inhibition of β-catenin target genes [13]. Following Wnt activation, GSK3β is phosphorylated allowing for β-catenin dissociation and translocation to the nucleus where it regulates the activity of genes governing self-renewal and proliferation [13, 14].

The Wnt/β-catenin signalling pathway is involved in the development and maintenance of AML LSCs, specifically enhancing the self-renewal and repopulation capacity of these cells [11, 12]. The expression levels of β-catenin are associated with poor patient prognosis and are correlated to drug resistance [15]. Common fusion products found in AML, such as AML1-ETO, MLL-AF9 and PML-RARα amplify canonical Wnt signaling in patient samples and β-catenin appears to be essential for progressing pre-LSCs into competent LSCs capable of long-term repopulation [16–19]. Importantly, impairment of β-catenin expression has demonstrated antileukemic properties, including the reversal of LSCs back to a pre-LSC like stage, reduced proliferation and sensitisation to targeted therapies [20].

NUC-7738 is a phosphoramidate transformation of the nucleoside 3’-deoxyadenosine (3’-dA), which is an adenosine derivative originally isolated from the fungus *Cordyceps sinensis*. 3’-deoxyadenosine triphosphate (3’-dATP), the active anti-cancer metabolite of 3’-dA, inhibits DNA and RNA synthesis, inducing apoptosis [21]. Although *in vitro* evidence suggests that 3’-dA (or cordycepin) may reduce β-catenin stability in leukemia by regulating GSK-3β and eliminating LSCs [22], the clinical utility is low. 3’-dA has not been successful in clinical studies, primarily due to the rapid degradation by adenosine deaminase (ADA). However, there has been promising efficacy of NUC-7738 in early clinical studies associated with activation of the drug leading to generation of 3’-dATP [21]. In this study, we aimed to determine whether NUC-7738 can reduce β-catenin signalling resulting in reduced proliferation and self-renewal of AML cells.

## Materials and methods

### Cell culture and reagents

Human OCI-AML3 and HL-60 cells were purchased from the American Type Culture Collection (ATCC) and U937 cells were provided by Dr Simon Powis (University of St Andrews, St Andrews, UK). They were maintained with/in RPMI 1640 (Gibco^TM^) supplemented with 10% FBS (Gibco^TM^), and 1% Penicillin/Streptomycin solution (Gibco^TM^). Human KG1a cells were provided by Professor Chris Pepper (Brighton and Sussex Medical School (BSMS), Brighton, UK) and were maintained with/in RPMI 1640 (Gibco^TM^) supplemented with 20% FBS (Gibco^TM^), and 1% Penicillin/Streptomycin solution (Gibco^TM^). Cell cultures were maintained in 5% CO2 at 37°C and were routinely tested to ensure that they were mycoplasma-free. All drug treatments were performed during the log phase of cell growth. All cells were tested to be Mycoplasma negative throughout the study.

NUC-7738 was provided as a powder by NuCana plc and was dissolved in 100% dimethyl sulfoxide (DMSO) (D4540, Sigma-Aldrich) to achieve a stock concentration of 10 mM, and stored as single use aliquots at -20°C.

### Cell apoptosis assay

AML cell lines were plated in 12 well plates at 2×10^5^ cells/well and treated with 5, 10 and 20 μM NUC-7738 or 0.2% DMSO for 48 hrs. Cells were transferred to 1.5 mL eppendorfs and washed twice with fresh cold FACS buffer (PBS, 0.5–1% BSA) at 200g centrifugation at 4°C for 5 minutes. Cells were then re-suspended in 100 μL of Annexin V Binding Buffer (422201, BioLegend) and 5 μL of Annexin-V conjugated to FITC (BioLegend, 640906). Cells were then vortexed and incubated at room temperature away from light for 15 minutes. An additional 100 μL of 3 μM DAPI (BioLegend, 422801) was added to each sample prior to flow cytometric analysis. Unstained and single stain controls were used to set appropriate gating and a stained sample spiked with 100 μL ethanol served as a positive control. Samples were analysed using the CytoFLEX V3-B4-R3 Flow Cytometer (Beckman Coulter).

### Western blot analysis

Cells were lysed with 1x CST lysis buffer (Cell Signalling Technologies, 9803) containing AEBSF protease inhibitor (Thermo Scientific, 78431), complete™ Mini EDTA-free protease inhibitor cocktail (Merck, 11836170001) and PhosSTOP™ (Merck, 4906845001). Following ice-cold PBS wash, cell pellets were lysed in 350 μL of lysis buffer and incubated for 10 minutes on ice. Lysates were vortexed, sonicated (Bioruptor® Pico) and then centrifuged at 14,000 x g for 10 minutes at 4°C. Supernatant was collected and total protein was determined using the Pierce™ BCA Protein Assay Kit (Thermo Scientific, 23225). Equal amounts of total protein from each sample were resolved by SDS-PAGE and transferred onto Nitrocellulose membranes (Amersham™ Protran®, GE10600002). Total protein was measured by staining the membrane with Revert™ 700 total protein stain (LI-COR, 926–11011) for protein normalisation. Membranes were then blocked with 3% BSA TBS-Tween (0.1%) and probed with the following antibodies; β-catenin (1:1000, Cell Signalling Technologies, 8480), c-Myc (1:1000, Cell Signalling Technologies, 5605), CD44 (1:1000, Cell Signalling Technologies, 3570), PI3 Kinase p110α (1:1000, Cell Signalling Technologies, 4249 phospho-Akt (Ser473) (1:1000, Cell Signalling Technologies, 4060) and phospho-GSK3β (Ser9) (1:1000, Cell Signalling Technologies, 9336). All primary antibodies used were raised in rabbit. Membranes were stained with IRDye® 800CW Goat anti-Rabbit secondary antibody (1:10,000 Abcam, ab216773) and membranes visualised using the Licor Odyssey scanner.

### Immunofluorescence

Following fixation with 4% formaldehyde, OCI-AML3 cells were embedded in 2% agarose gel and processed in Leica tissue processor ASP300S overnight, which included dehydration, clearing, and infiltration using alcohols, xylenes, and paraffin, respectively. Agarose embedded cells were then embedded in paraffin. Formalin Fixed paraffin embedded (FFPE) cell blocks were cut at 2.5 μm. The sections were then haematoxylin and eosin stained. Tissue microarray (TMA) was constructed using 1 mm diameter recipient punch in a recipient block. The TMA section was cut at 2.5 μm thickness. Immunofluorescence was performed using Leica Bond RX autostainer. Section was dewaxed and rehydrated in Bond dewax solution (Leica, MSPP-AR9222) and absolute alcohol. Epitope retrieval buffer 1 (Leica, AR9961) was used to unmask the epitopes. 3% Hydrogen peroxide was used to block endogenous peroxidase activity, followed by serum free protein block to quench non-specific background. Primary antibody (β-catenin, BD Bioscience, 610153) was incubated for 40 minutes, followed by post primary and polymer secondary antibody incubation from Leica Bond polymer refined detection kit (Leica, DS9800). Primary antibody was visualised by fluorescein conjugated tyramide signal amplification (Akoya bioscience, NEL741001KT). Sections were counterstained using Hoechst 33342 (Thermo fisher, H3570) and mounted in prolong gold anti-fade mounting medium (Thermo fisher, P36930). Image was captured in Zeiss Axio scan z1 under 40x object magnification. Hoechst and FITC channels were applied to capture images with 25 and 40 milliseconds exposure time. Indica HALO AI was utilised to quantify FITC intensity in an individual cell.

### Characterization of CD34^+^CD38^-^CD123^+^ LSC-like cells

KG1a cells were plated in 6 well plates and 1×10^6^ cells per well were treated with 5, 10 and 20 μM NUC-7738 for 48 hrs. Cells were washed twice with ice-cold FACS buffer (0.5–1% BSA) and resuspended in FACS buffer containing 2.5 μL of Human TruStain FcX™ (Biolegend, 422302) and cells were incubated for 10 minutes at room temperature. Cells were washed twice with FACS buffer and stained with 1.5uL of FITC anti-CD34 (Biolegend, 343604), PE anti-CD38 (Biolegend, 102718) and anti-CD123 (Biolegend, 306006). Unstained, single stained and fluorescence-minus-one controls (FMOs) were used for fluorescence compensation and accurate gating.

### Colony forming assay

HL-60 cells were treated with NUC-7738 for 72 hrs prior to seeding 30 x 10^3^ cells onto MethoCult^TM^ enriched medium (Stemcell Technologies, UK, H4435) and colonies were recorded using Celigo (Nexcelom Bioscience) after 14 days incubation at 37°C and 5% CO2 incubation.

### Statistical analysis

GraphPad Prism software (v9) was used when performing statistical analysis and in the creation of graphs. Where the unpaired Student’s t-test was performed for statistical analysis: **** for P<0.0001, *** for P<0.001, ** for P<0.01 and * for P<0.05 were considered statistically significant and were indicated in the related figure legends and graphs. Where n.s. is indicated, the P value is not significant. The results are shown as mean ± standard deviation (SD). CytoFlex software (Beckman Coulter) was used when performing analysis of flow cytometric data. QuPath: Open source software for digital pathology image analysis was used to perform colony quantification [23]. Empiria Studio® Software (LI-COR) was used to quantify western blot bands and normalise bands to total protein.

## Results

### NUC-7738 causes apoptosis and reduces the viability of AML cells

**Fig 1:**
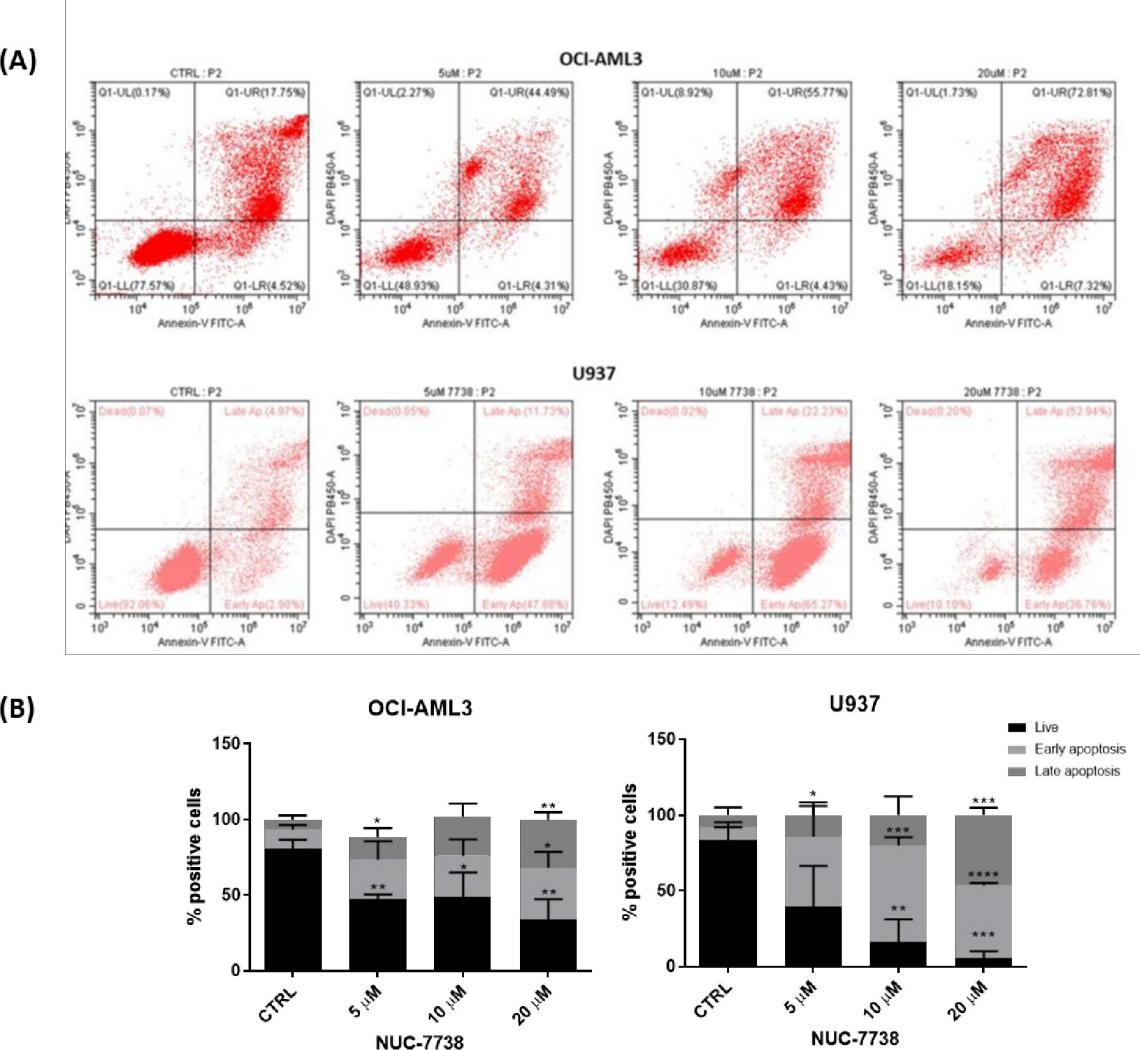
NUC-7738 induces apoptosis in AML cell lines. OCI-AML3 and U937 cells were treated with NUC-7738 at 5, 10 and 20 μM for 48 hrs and apoptosis was determined by Annexin-V DAPI staining. (A) dot plots generated during flow cytometry analysis highlighting the percentage of live (LL), early apoptotic (LR), late apoptotic (UR) and dead (UL) cells. (B) Bar graphs highlight the proportion of cells which are viable, in early apoptosis or in late apoptosis as determined by Annexin-V / DAPI staining, and analysis by flow cytometry. The percentages shown indicate the proportion of total events within each of the respective categories as gated on the flow cytometry plots. Gating strategy highlighted in S1 Fig in S1 File. Each bar represents the mean percentage from three independent experiments with error bars indicating SD showing NUC-7738 caused a reduction in viable cells and an increase in early and late apoptotic cells in all cells examined. NUC-7738 did not induce apoptosis in the KG1a cell line (S20 Fig in S1 File).

To understand whether NUC-7738 can induce apoptosis in AML cells, OCI-AML3 and U937 cells were treated with 5, 10 and 20 μM of NUC-7738 for 48 hours. Cell apoptosis was determined by staining cells with Annexin-V and DAPI, followed by flow cytometry assessment. The percentage of early apoptotic control cells increased from 8.3% to 63.5% in U937 cells treated with 10 μM which was followed by a further increase in late apoptotic cells from 8.2% to 46.4% in the 20 μM treated arm. This trend in early and late apoptotic cells was also apparent for the OCI-AML3 cell line (Fig 1).

### NUC-7738 inhibits β-catenin signalling

**Fig 2:**
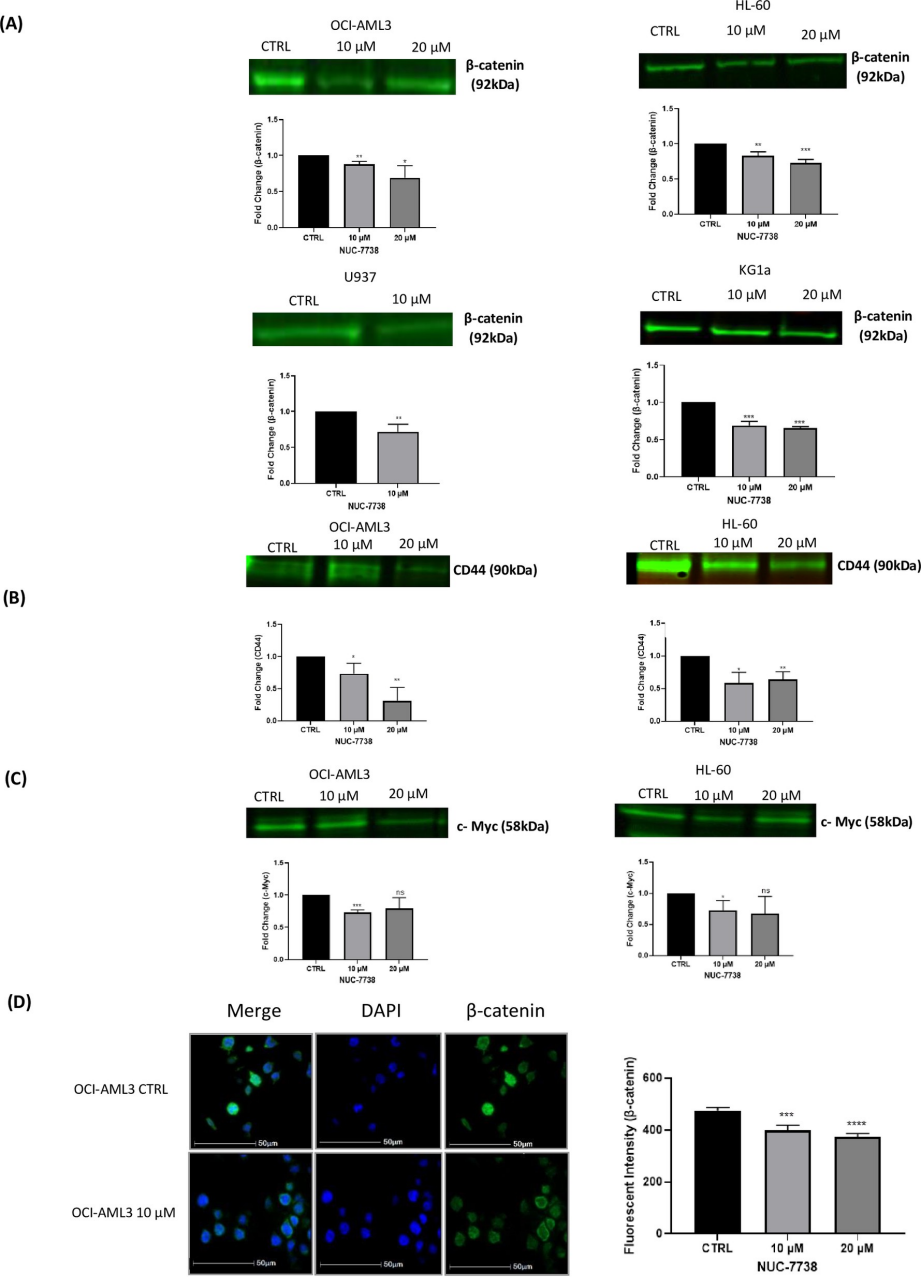
NUC-7738 inhibits β-catenin and its target genes c-Myc and CD44. OCI-AML3, HL-60 and KG1a cells were treated with NUC-7738 at 10 and 20 μM for 48 hrs, U937 cells were only treated with 10 μM. β-catenin (A), c-Myc (B) and CD44 (C) protein levels were determined by Western blot analysis and bands were normalised to total protein. Protein levels of β-catenin, c-Myc and CD44 were all reduced in a dose-dependent manner in all tested cell lines following 48 hrs NUC-7738 treatment. (D) β-catenin was localised using immunofluorescence, highlighting the non-nuclear localisation of β-catenin (white arrow) following 48 hrs treatment with NUC-7738 in OCI-AML3 and the reduction of total β-catenin. These data are from three independent experiments. Each bar denotes mean ± SD * P<0.05, ** P<0.01, **** P< 0.001.

Due to the WNT pathway being involved in the development and progression of AML, we questioned whether the cell death inducing properties of NUC-7738 were due to alteration of β-catenin signalling. OCI-AML3, HL-60 and KG1a cells were treated with 10 and 20 μM NUC-7738, whereas U937 cells were only treated with 10 μM NUC-7738 for 48 hrs and expression of β-catenin was determined by Western blotting. CD44 and c-Myc expression was determined by Western blotting for OCI-AML3 and HL-60 cells following 48 hours treatment with 10 and 20 μM NUC-7738. At 20 μM of treatment, there was a 31%, 29% and 35% significant decrease in β-catenin protein levels in OCI-AML3, HL-60 and KG1a cells, respectively (Fig 2A). There was also a 69% and 45% significant decrease in CD44 protein levels in OCI-AML3 and HL-60 cells, respectively at 20 μM (Fig 2B). The protein levels of c-Myc were also significantly decreased by 28% in both OCI-AML3 and HL-60 cells at 10 μM (Fig 2C). Overall, NUC-7738 significantly decreased the protein levels of β-catenin, c-Myc and CD44 in all tested cell lines (Fig 2A–2C). As the biochemical hallmark of WNT pathway activation is expression of nuclear β-catenin, we performed immunofluorescence detection of β-catenin in OCI-AML3 cells. Following 48 hrs treatment of OCI-AML3 cells with 10 and 20 μM NUC-738, both total and nuclear β-catenin expression was significantly suppressed, indicating inhibition of this pathway (Fig 2D).

### NUC-7738 reduces the self-renewal of AML cells

**Fig 3:**
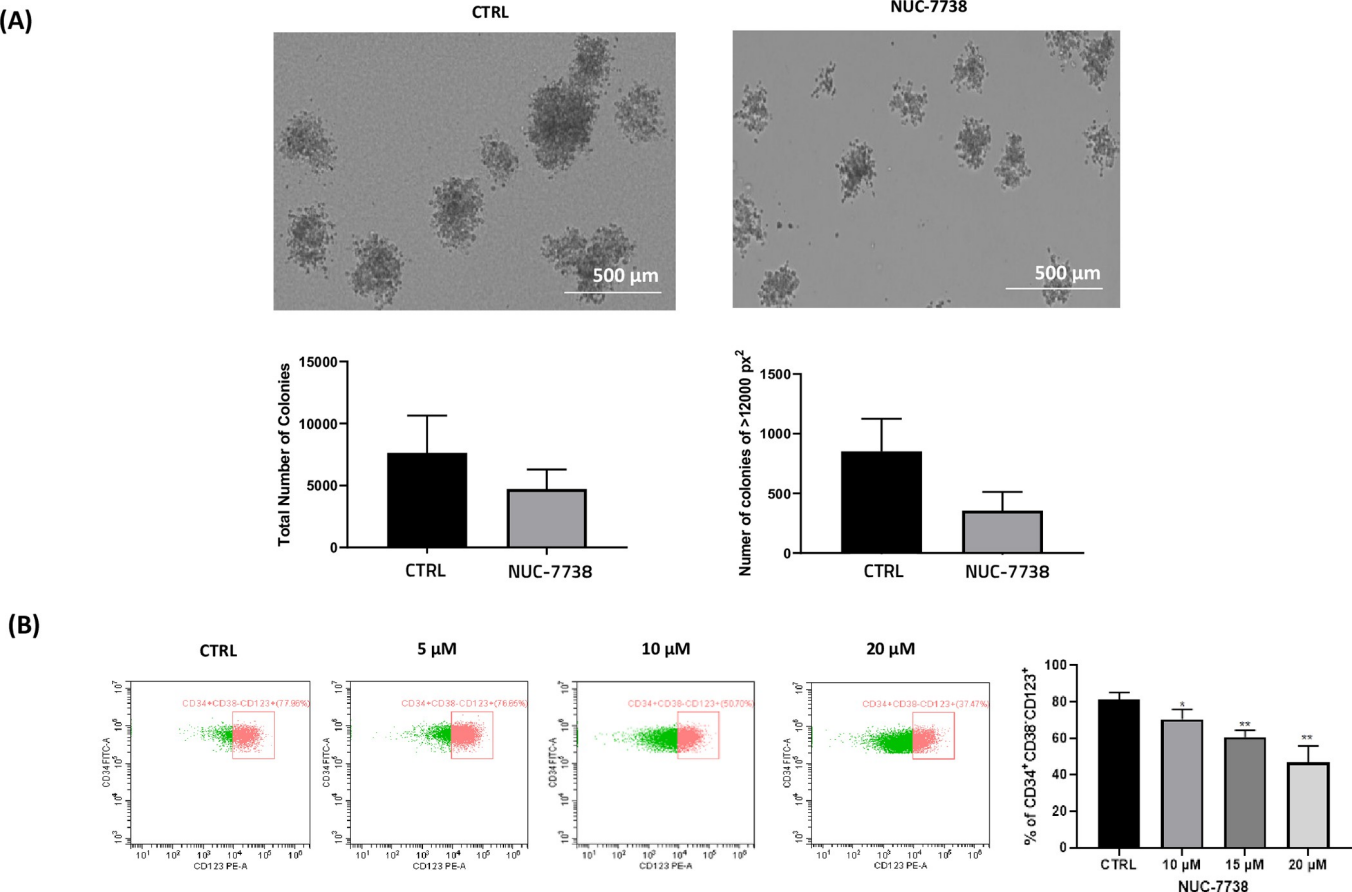
NUC-7738 reduces colony formation and LSC-like cells in leukemia (A) HL-60 cells were treated with NUC-7738 for 72 hours followed by plating 30 x 10^3^ cells onto MethoCult medium and recording colonies after 14 days. The total number and size of colonies were reduced following NUC-7738 treatment (B) KG-1a cells were treated with 5, 10 and 20 μM NUC-7738 for 48 hours followed by cell surface staining for CD34, CD38 and CD123, in addition to DAPI staining to negate dead cells. These data are from three independent experiments. Each bar denotes mean ± SD * P<0.05, ** P<0.01.

Due to the key role that β-catenin has in the maintenance of AML self-renewal and survival, we examined the effects of NUC-7738 on the colony forming capability of leukemia cells, in addition to measuring the percentage of LSC-like cells (CD34^+^CD38^-^CD123^+^). HL-60 cells were treated for 72 hrs with NUC-7738 prior to being seeded onto MethoCult media. KG1a cells were treated with 10, 15 and 20 μM NUC-7738 for 48 hrs prior to flow cytometry assessment of the percentage of CD34^+^CD38^-^CD123^+^ cells. NUC-7738 also reduced the number and size of leukemic colonies, in addition to changing colony morphology (Fig 3A). The percentage of CD34^+^CD38^-^CD123^+^ cells were significantly reduced in a dose dependant manner, reducing from 81% to 71%, 61% and 47% at 10, 15 and 20 μM, respectively. (Fig 3B). These results indicate that NUC-7738 can inhibit the survival and renewal of LSCs.

### NUC-7738 regulates β-catenin through PI3K/Akt/GSK3β signalling

After establishing that NUC-7738 reduces β-catenin signalling which in turn affects stemness of AML cells, we investigated the mechanisms underlying the regulation of β-catenin. It is recognised that the PI3K/AKT signalling cascade is involved in the excessive proliferation and survival of AML cells and that this pathway, through inhibition of GSK3β, can activate β-catenin. We hypothesised that NUC-7738 inhibits PI3K/Akt resulting in GSK3β mediated phosphorylation and subsequent proteasomal degradation of β-catenin. To investigate this, HL-60 and OCI-AML3 cells were treated with 10 and 20 μM NUC-7738 for 48 hrs, whereas the U937 cells were treated with 10 μM only. This was followed by the assessment of the protein levels of PI3K-p110α, phosphorylated Akt (Ser473) and phosphorylated GSK3β (Ser9) by Western blotting.

**Fig 4:**
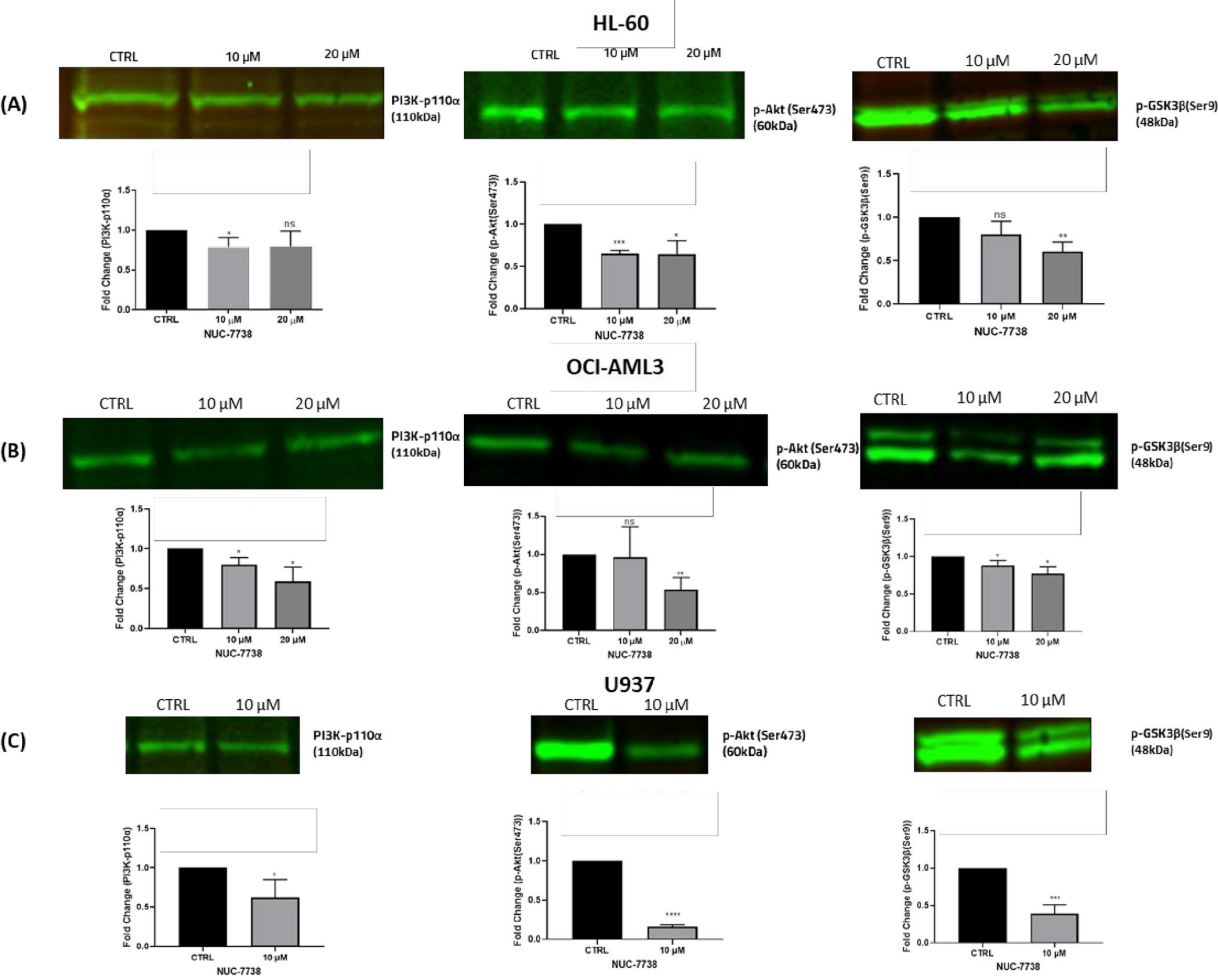
PI3K/Akt/MAPK signaling is involved in NUC-7738 mediated suppression of β-catenin. (A) HL-60, (B) OCI-AML3 were treated with 10 and 20 μM NUC-7738, whereas (C) U937 cells were only treated with 10 μM for 48 hrs. Expression level of PI3K-p110α, phosphorylated Akt (Ser473) and phosphorylated GSK3β (Ser9) was determined by Western blot analysis and bands normalised to total protein. In each cell line tested there was >40% reduction in the protein levels of phosphorylated Akt (Ser473) and phosphorylated GSK3β (Ser9), following 48 hrs NUC-7738 treatment.

**Fig 5:**
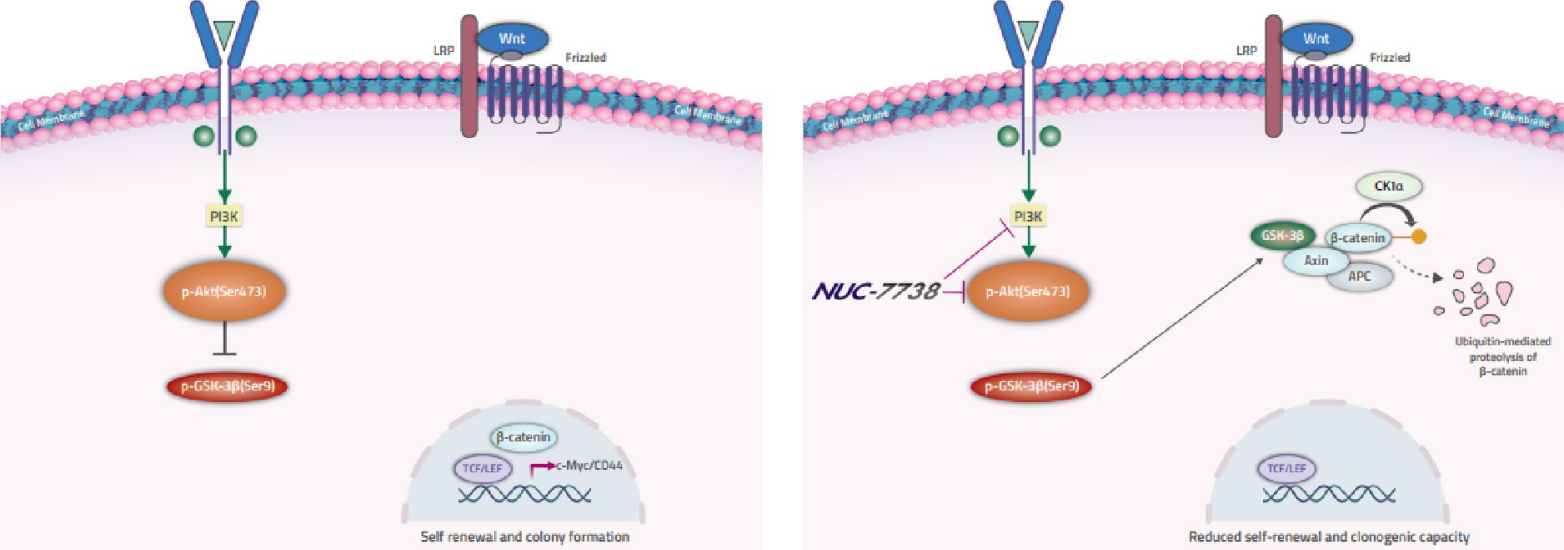
A schematic model illustrating the proposed role of NUC-7738 in suppressing β-catenin in leukemia cells.

NUC-7738 significantly reduced the protein levels of PI3K-p110α and phosphorylated GSK3β (Ser9) in all three cell lines (Fig 4A–4C). In OCI-AML3 and HL-60 cells at 20 μM, there was a 46% and 36% significant reduction in phosphorylated Akt (Ser473) protein levels, respectively (Fig 4A and 4B). The largest significant changes occurred in the U937 cell line, which exhibited a 84% and 56% decrease in phosphorylated Akt (Ser473) and phosphorylated GSK3β (Ser9) protein levels at 10 μM, respectively (Fig 4C). The differences in the reduced expression of these proteins can be correlated to the sensitivities of the different AML cell lines to NUC-7738. These results suggest that NUC-7738 may regulate β-catenin signalling through removing the inhibitory effect of PI3K/Akt on GSK3β resulting in GSK3β-mediated ubiquitination and degradation of β-catenin (Fig 5).

## Discussion

The presence of non-GSK3β-bound β-catenin predominantly in the nucleus is recognised as being important in the pathogenesis of AML and patient prognosis, highlighting the clinical potential of targeting the Wnt pathway [15, 24–28]. Additionally, β-catenin activation is implicated in one of the most common AML mutations, the FLT3–ITD mutation [28]. We demonstrated that NUC-7738 induces apoptosis in OCI-AML3 and U937 cells following 48 hours treatment. This was further confirmed by the dose-dependent increase in cleaved caspase 3 positivity following NUC-7738 treatment of AML cells (S2 Fig in S1 File). Due to the key role that the β-catenin signalling pathway has in the maintenance of AML, specifically the LSCs, we investigated whether the cytotoxic effect of NUC-7738 was due to inhibition of the β-catenin pathway.

NUC-7738 caused a dose-dependent reduction in the protein levels of β-catenin in OCI-AML3, HL-60 and U937 cells following 48 hours treatment. In order to assess whether the regulation of β-catenin altered the translation of its target genes, CD44 and c-Myc protein levels were assessed. Both, CD44 and c-Myc play crucial roles in driving leukemogenesis [17, 29, 30]. CD44 is overexpressed in AML and is involved in chemoresistance, whereas overactivation of c-Myc can induce AML in mice [29, 31, 32]. NUC-7738 significantly reduced the expression of CD44 in both OCI-AML3 and HL-60 cells, suggesting that not only does the inhibition of β-catenin by NUC-7738 result in inhibition of its downstream target CD44, but also that NUC-7738 could potentially inhibit homing of AML cells to microenvironmental niches and relieve the primitive phenotypes of such cells. NUC-7738 also significantly reduced the protein levels of c-Myc, which may suggest that NUC-7738 could provide anti-leukemic activity in patients expressing RUNX1-ETO, PML-RARα, and PLZF-RARα chromosomal translocations as these all induce c-Myc expression [17, 31, 33]. As the ‘hallmark’ of β-catenin activation is its localization to the nucleus, NUC-7738-mediated reduction of nuclear β-catenin provides confirmation that NUC-7738 inhibits β-catenin signalling. We acknowledge that future studies should also quantify cytoplasmic levels of β-catenin, as this, combined with identifying phosphorylated and ubiquitinated β-catenin, would allow for the rate of β-catenin cytosolic destruction to be assessed.

Given the important role of β-catenin signalling in the survival and maintenance of LSCs, we examined the effect of NUC-7738 on the colony forming capability of AML cells and the proportion of LSC-like cells. NUC-7738 reduced the number and size of leukemic colonies and the percentage of CD34^+^CD38^-^CD123^+^ LSCs. These results correlate with the importance of β-catenin regulated self-renewal of AML cells and illustrate that NUC-7738 suppresses β-catenin regulation in leukemia cells resulting in reduced clonogenicity.

There is frequent upregulation of the PI3K/Akt pathway in AML, which corresponds to a worse clinical outcome [34, 35]. In addition, complete activation of Akt has been observed in approximately 70% of AML patients [34, 35]. As unphosphorylated GSK3β is involved in preventing β-catenin activation and phosphorylated Akt (Ser473) relieves GSK3β-mediated inhibition of β-catenin, the effect of NUC-7738 on the PI3K/Akt pathway was investigated. Indeed, NUC-7738 significantly reduced the protein levels of PI3K-p110α, phosphorylated Akt (Ser473) and phosphorylated GSK3β (Ser9) in OCI-AML3, HL-60 and U937cells. These results suggest that NUC-7738 may regulate β-catenin signalling through removal of the inhibitory effect of PI3K/Akt on GSK3β resulting in GSK3β-mediated ubiquitination and degradation of β-catenin, highlighting that NUC-7738 may also be effective in patients with enhanced tyrosine kinase signalling.

Since normal adult haematopoietic stem cells (HSCs) do not require β-catenin for survival or self-renewal [13] and β-catenin can be highly expressed in AML patients [13, 14], therapeutic targeting of β-catenin poses an attractive option for treatment. Indeed, several pharmaceutical product candidates targeting the Wnt pathway are under development, some of which are undergoing Phase I/2 clinical assessment [35, 36]. Proteomic studies have revealed for the first time that β-catenin also interacts with Wilms tumour protein (WT1), in an RNA-independent manner. Interestingly, these studies demonstrated that β-catenin also co-localised with WT1 in the nucleus during Wnt activation [37, 38]. Since this was confirmed in the HL-60 cells, in addition to primary samples, and that we have shown inhibition of β-catenin in multiple AML cell lines, it would be interesting to distinguish whether NUC-7738 also inhibits WT1 and whether this heightens the anti-leukemic response. This could be clinically important due to the non-discriminatory expression of the β-catenin-WT1 interactome regarding AML genotype and the fact that WT1 mutations are known to be associated with leukemogensis [39]. Intricate *in vivo* studies by the So group have linked restoring the anti-cancer effect of GSK3 inhibitors with reduction in β-catenin expression in MLL-transformed cells [16]. Since we believe that NUC-7738 can also restore the β-catenin inhibitory effect of GSK3β by reducing its phosphorylation on serine 9 by Akt, it would be important to validate whether NUC-7738 could also reverse LSCs into a pre-LSC state as has been shown by β-catenin inhibition in MLL cells.

Overall, our findings indicate the NUC-7738 inhibits β-catenin signalling through GSK3β-mediated degradation of β-catenin, inhibition of β-catenin target genes and reduction in the self-renewal of AML cells resulting in eventual apoptosis. These findings, combined with a favorable safety profile in the clinic to date [21], provide support for evaluating NUC-7738 for the treatment of patients with AML.

## Supporting information

S1 File (PDF)
