# Supplementary material for: NUC-7738 regulates β-catenin signalling resulting in reduced proliferation and self-renewal of AML cells
Source: PLoS One. 2022 Dec 15;17(12):e0278209. doi: 10.1371/journal.pone.0278209 (PMC9754587; doi:10.1371/journal.pone.0278209)
Supplement: S1 File — (PDF) [file pone.0278209.s001.pdf]

# Supplementary

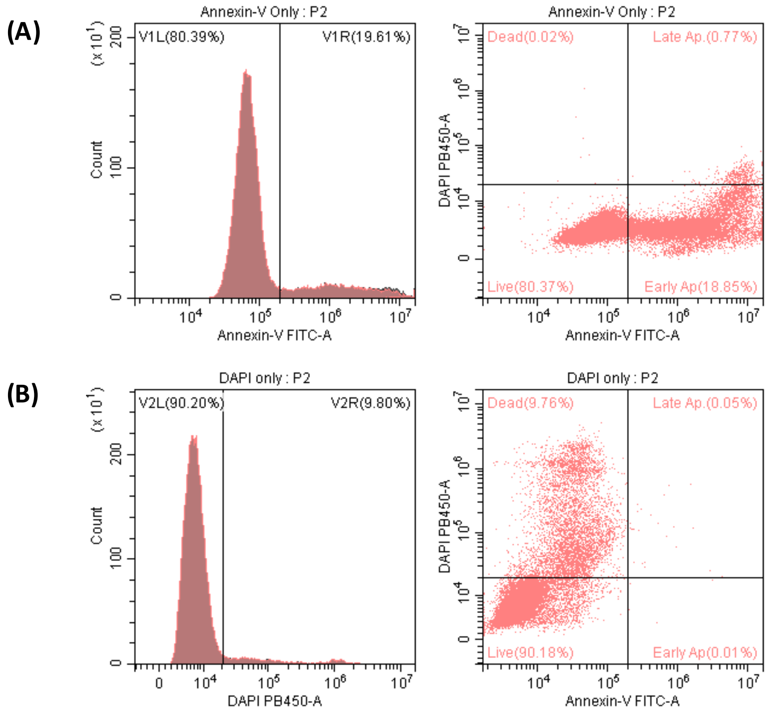


**Supplementary Figure 1.** Gating strategy to determine apoptotic cells. Cells were either stained with Annexin-V only (A) or DAPI only (B) to generate single stain controls which were used to determine gating for live, early apoptotic, late apoptotic and dead cells.

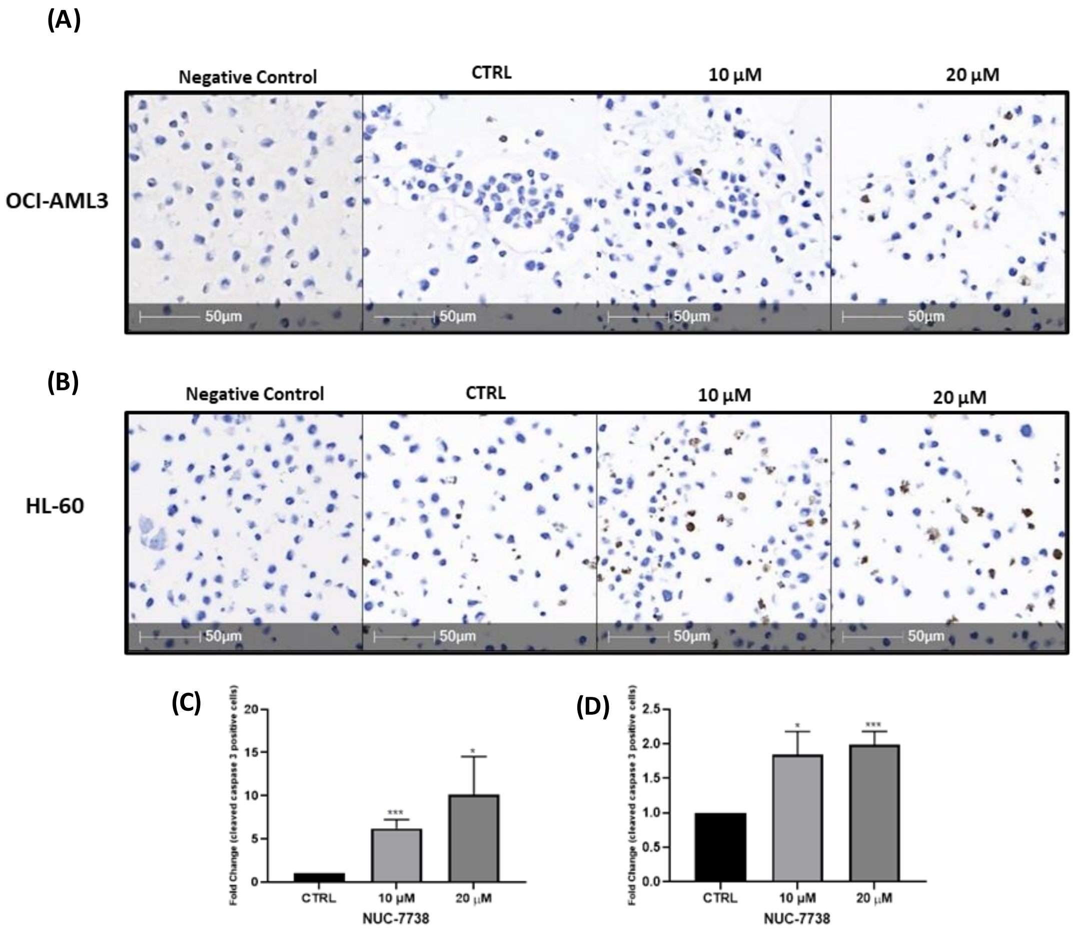


**Supplementary Figure 2**. Immunohistochemistry (IHC) for cleaved caspase 3 positive cells following culture of OCI-AML3 and HL-60 cells with select concentrations of NUC-7738. Representative IHC brown staining for cleaved caspase 3 positive cells in OCI-AML3 (40x magnification) (A) and HL-60 (40x magnification) (B) cells, following 48 hrs treatment with 10 and 20 µM NUC-7738. Positive signal detection on IHC was based on secondary only controls (negative control). Graphs indicate the fold change in the number of cleaved caspase positive cells as determined by image analysis using Indica HALO AI software for OCI-AML3 (C) and HL-60 (D). Each bar represents the mean percentage from three independent experiments with error bars indicating SD. Statistically significant results are highlighted with p value to the nearest 3 decimal places. Data are represented by mean ± SD. Student's t-test was used to compare the groups; ****$p<0.0001$ ***$p<0.001$, **$p<0.01$, *$p<0.05$. This data demonstrates the clear increase in the proportion of cleaved caspase 3 positive cells in both OCI-AML3 and HL-60 cells following 48 hrs NUC-7738 treatment, an indication of the final stage of apoptosis.

(A)

β-catenin
(92kDa)

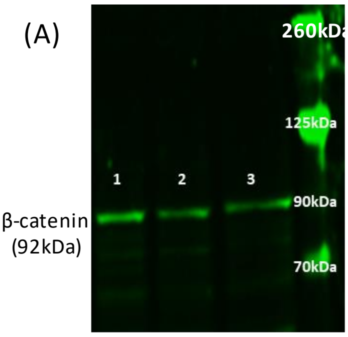


(B)

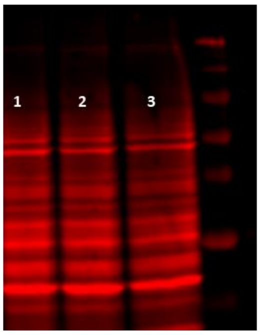


| CTRL | 10 μM | 20 μM |
|---|---|---|
| 1 | 0.925003 | 0.803803 |
| 1 | 0.856333 | 0.499707 |
| 1 | 0.84669 | 0.77359 |

**Supplementary Figure 3.** β-catenin expression in OCI-AML3 cells following 48 hours treatment with NUC-7738. Full Western blot representative of one biological repeat, highlighting β-catenin protein expression (A) and total protein expression (B) for each sample. 1, OCI-AML3 CTRL; 2, OCI-AML3 10 μM; OCI-AML3 20 μM. Table illustrates the quantified fold changes which were used to generate the OCI-AML3 β-catenin expression graphs in Figure 2A, after normalising to total protein.

(A)

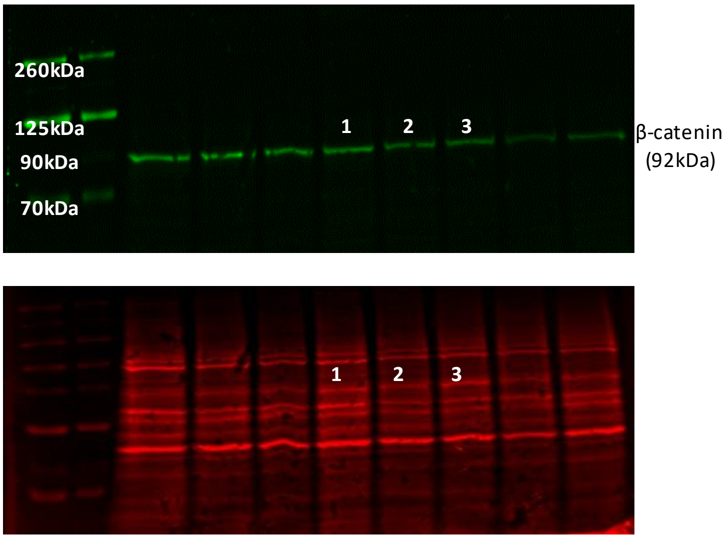


| CTRL | 10 µM | 20 µM |
|---|---|---|
| 1 | 0.830618 | 0.775633 |
| 1 | 0.885722 | 0.729459 |
| 1 | 0.766724 | 0.672802 |

**Supplementary Figure 4.** β-catenin expression in HL-60 cells following 48 hours treatment with NUC-7738. Full Western blot representative of one biological repeat, highlighting β-catenin protein expression (A) and total protein expression (B) for each sample. 1, HL-60 CTRL; 2, HL-60 10 µM; HL-60 20 µM. Table illustrates the quantified fold changes which were used to generate the HL-60 β-catenin expression graphs in Figure 2A, after normalising to total protein.

(A)

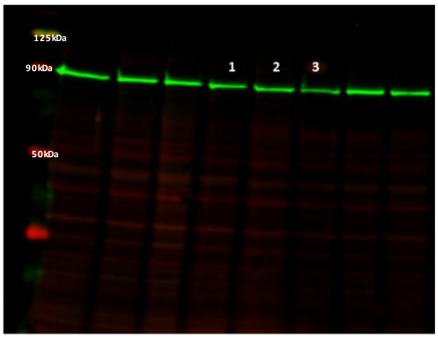


β-catenin
(92kDa)

(B)

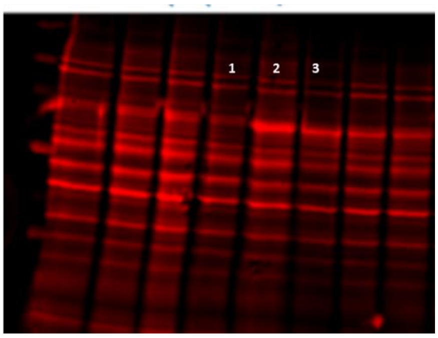


| CTRL | 10 µM | 20 µM |
|---|---|---|
| 1 | 0.724855 | 0.669357 |
| 1 | 0.719975 | 0.6845 |
| 1 | 0.625721 | 0.634511 |

**Supplementary Figure 5.** β-catenin expression in KG1a cells following 48 hours treatment with NUC-7738. Full Western blot representative of one biological repeat, highlighting β-catenin protein expression (A) and total protein expression (B) for each sample. 1, KG1a CTRL; 2, KG1a 10 µM; KG1a 20 µM. Table illustrates the quantified fold changes which were used to generate the KG1a β-catenin expression graphs in Figure 2A, after normalising to total protein.

(A)

(B)

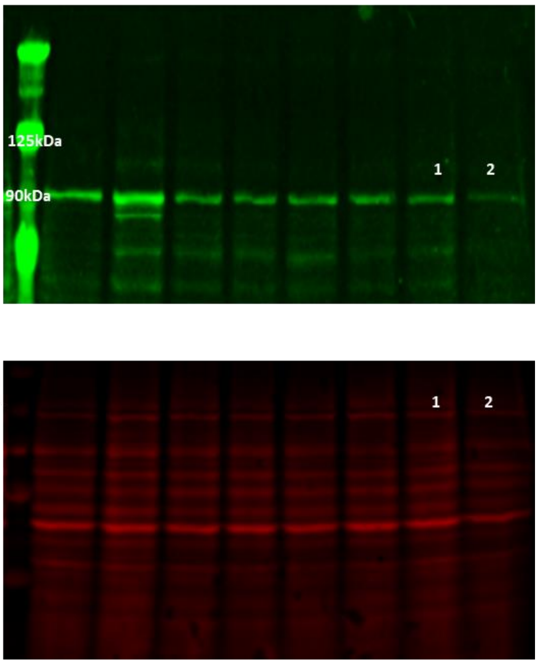


β-catenin
(92kDa)

| CTRL | 10 µM |
|---|---|
| 1 | 0.814328 |
| 1 | 0.731381 |
| 1 | 0.605101 |

**Supplementary Figure 6.** β-catenin expression in U937 cells following 48 hours treatment with NUC-7738. Full Western blot representative of one biological repeat, highlighting β-catenin protein expression (A) and total protein expression (B) for each sample. 1, U937 CTRL; 2, U937 10 µM; U937 20 µM. Table illustrates the quantified fold changes which were used to generate the U937 β-catenin expression graphs in Figure 2A, after normalising to total protein.

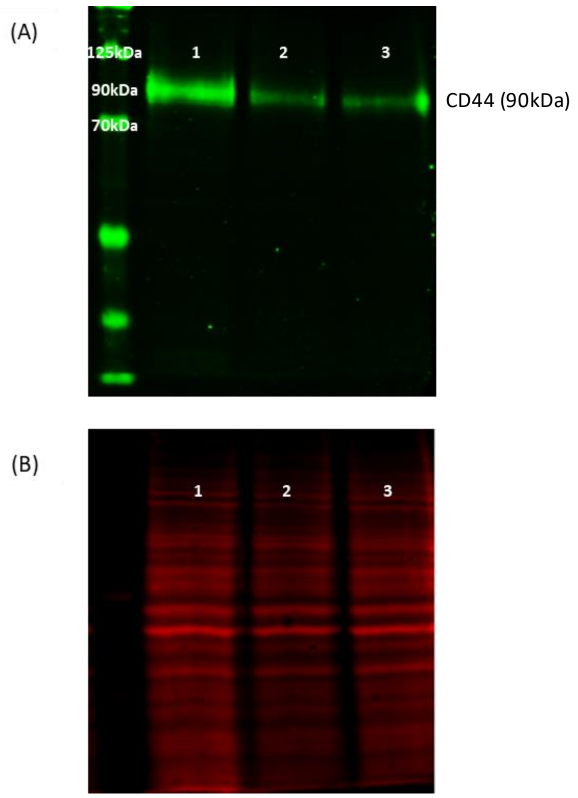


| CTRL | 10 µM | 20 µM |
|---|---|---|
| 1 | 0.501191 | 0.425026 |
| 1 | 0.724395 | 0.567179 |
| 1 | 0.754568 | 0.686422 |

**Supplementary Figure 7.** CD44 expression in HL-60 cells following 48 hours treatment with NUC-7738. Full Western blot representative of one biological repeat, highlighting CD44 protein expression (A) and total protein expression (B) for each sample. 1, HL-60 CTRL; 2, HL-60 10 µM; HL-60 20 µM. Table illustrates the quantified fold changes which were used to generate the HL-60 CD44 expression graphs in Figure 2B, after normalising to total protein.

(A)

CD44 (90kDa)

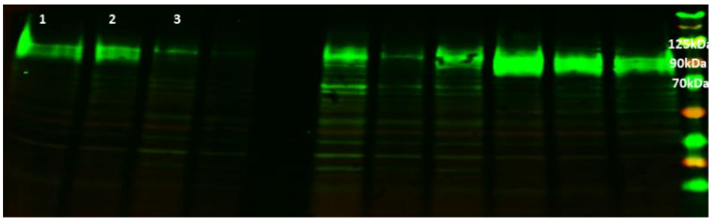


(B)

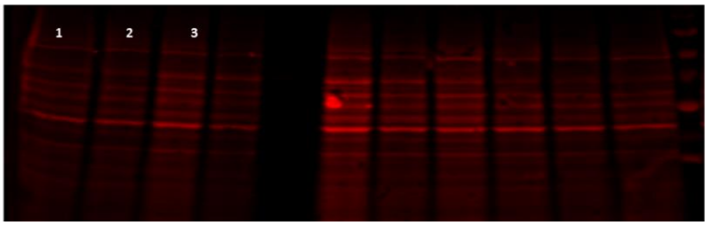


| CTRL | 10 µM | 20 µM |
|---|---|---|
| 1 | 0.647304 | 0.110517 |
| 1 | 0.640991 | 0.526632 |
| 1 | 0.9218 | 0.302239 |

**Supplementary Figure 8.** CD44 expression in OCI-AML3 cells following 48 hours treatment with NUC-7738. Full Western blot representative of one biological repeat, highlighting CD44 protein expression (A) and total protein expression (B) for each sample. 1, OCI-AML3 CTRL; 2, OCI-AML3 10 µM; OCI-AML3 20 µM. Table illustrates the quantified fold changes which were used to generate the OCI-AML3 CD44 expression graphs in Figure 2B, after normalising to total protein.

(A)

c-Myc (58kDa)

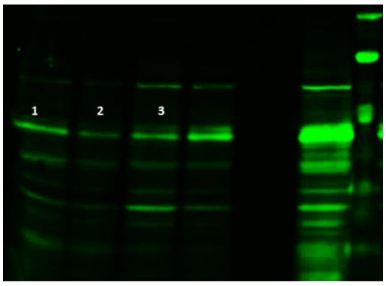


(B)

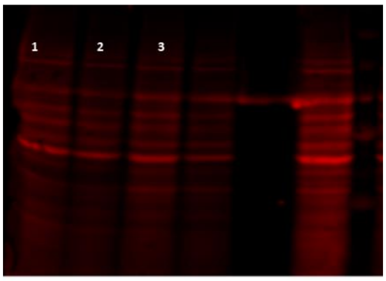


| CTRL | 10 µM | 20 µM |
|---|---|---|
| 1 | 0.559462 | 0.773597 |
| 1 | 0.73858 | 0.885608 |
| 1 | 0.876979 | 0.350788 |

**Supplementary Figure 9.** c-Myc expression in HL-60 cells following 48 hours treatment with NUC-7738. Full Western blot representative of one biological repeat, highlighting c-Myc protein expression (A) and total protein expression (B) for each sample. 1, HL-60 CTRL; 2, HL-60 10 µM; HL-60 20 µM. Table illustrates the quantified fold changes which were used to generate the HL-60 c-Myc expression graphs in Figure 2C, after normalising to total protein.

(A)

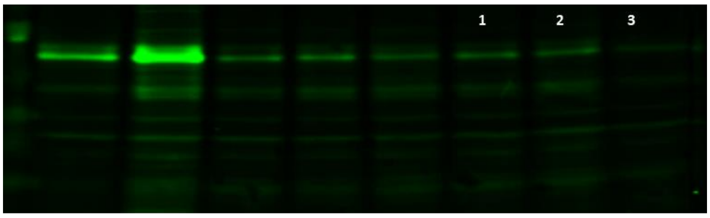


c-Myc (58kDa)

(B)

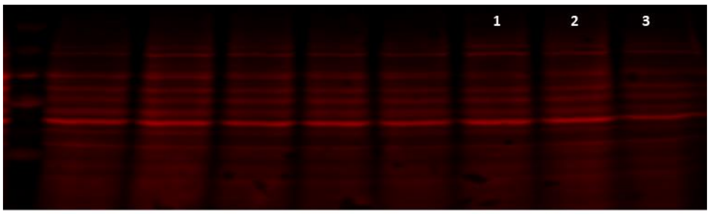


| CTRL | 10 µM | 20 µM |
|---|---|---|
| 1 | 0.76313 | 0.890056 |
| 1 | 0.679146 | 0.589102 |
| 1 | 0.742348 | 0.885833 |

**Supplementary Figure 10.** c-Myc expression in OCI-AML3 cells following 48 hours treatment with NUC-7738. Full Western blot representative of one biological repeat, highlighting c-Myc protein expression (A) and total protein expression (B) for each sample. 1, OCI-AML3 CTRL; 2, OCI-AML3 10 µM; OCI-AML3 20 µM. Table illustrates the quantified fold changes which were used to generate the OCI-AML3 c-Myc expression graphs in Figure 2B, after normalising to total protein.

(A)

(B)

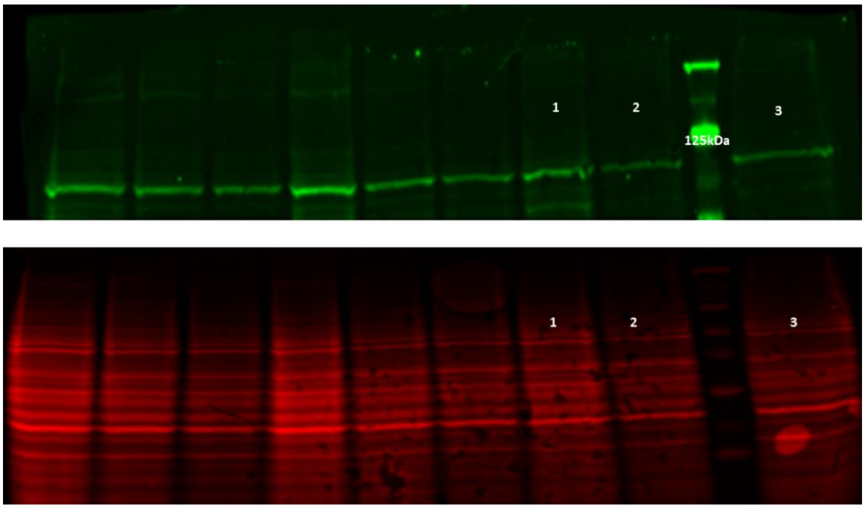


PI3K-p110α (110kDa)

| CTRL | 10 µM | 20 µM |
|---|---|---|
| 1 | 0.891713 | 1.009006 |
| 1 | 0.821303 | 0.74053 |
| 1 | 0.65838 | 0.635841 |

**Supplementary Figure 11.** PI3K-p100α expression in HL-60 cells following 48 hours treatment with NUC-7738. Full Western blot representative of one biological repeat, highlighting PI3K-p100α protein expression (A) and total protein expression (B) for each sample. 1, HL-60 CTRL; 2, HL-60 10 µM; HL-60 20 µM. Table illustrates the quantified fold changes which were used to generate the HL-60 PI3K-p100α expression graphs in Figure 4A, after normalising to total protein.

(A)

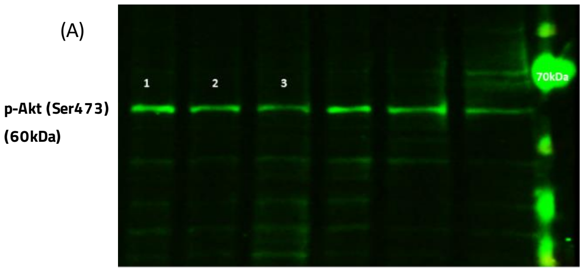


(B)

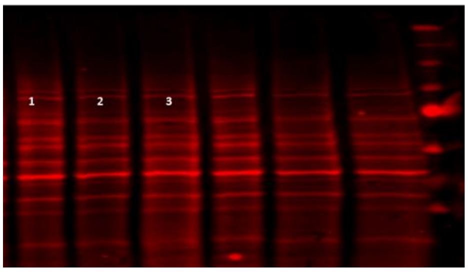


| CTRL | 10 µM | 20 µM |
|---|---|---|
| 1 | 0.624351148 | 0.543030307 |
| 1 | 0.697450952 | 0.562900858 |
| 1 | 0.617787099 | 0.82683498 |

**Supplementary Figure 12.** p-Akt (Ser473) expression in HL-60 cells following 48 hours treatment with NUC-7738. Full Western blot representative of one biological repeat, highlighting p-Akt (Ser473) protein expression (A) and total protein expression (B) for each sample. 1, HL-60 CTRL; 2, HL-60 10 µM; HL-60 20 µM. Table illustrates the quantified fold changes which were used to generate the HL-60 p-Akt (Ser473) expression graphs in Figure 4A, after normalising to total protein.

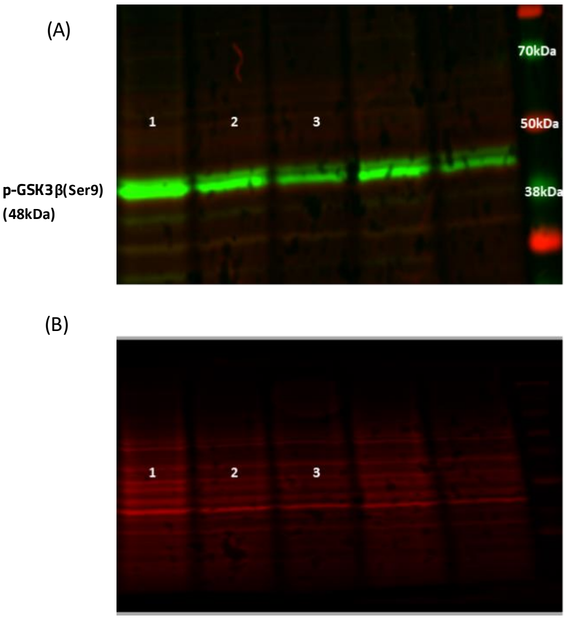


| CTRL | 10 µM | 20 µM |
|---|---|---|
| 1 | 0.963714 | 0.680731 |
| 1 | 0.779049 | 0.480041 |
| 1 | 0.667862 | 0.651321 |

**Supplementary Figure 13.** p-GSK3β(Ser9) expression in HL-60 cells following 48 hours treatment with NUC-7738. Full Western blot representative of one biological repeat, highlighting p-GSK3β(Ser9) protein expression (A) and total protein expression (B) for each sample. 1, HL-60 CTRL; 2, HL-60 10 µM; HL-60 20 µM. Table illustrates the quantified fold changes which were used to generate the HL-60 p-GSK3β(Ser9) expression graphs in Figure 4A, after normalising to total protein.

(A)

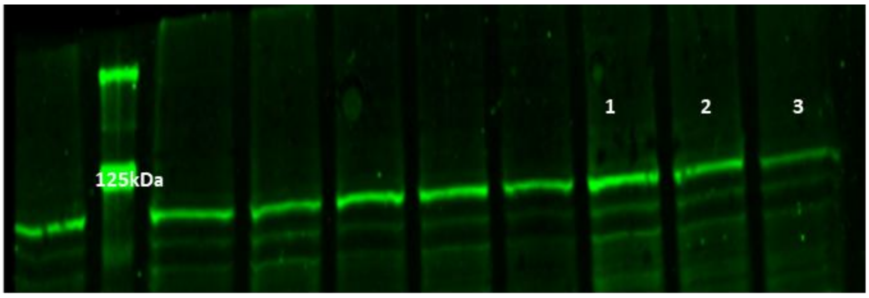


PI3K-p110α (110kDa)

(B)

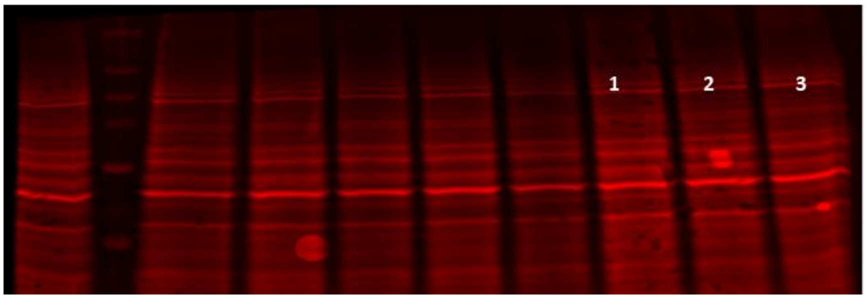


| CTRL | 10 µM | 20 µM |
|---|---|---|
| 1 | 0.877153 | 0.546568 |
| 1 | 0.815801 | 0.786872 |
| 1 | 0.703968 | 0.422261 |

**Supplementary Figure 14.** PI3K-p110α expression in OCI-AML3 cells following 48 hours treatment with NUC-7738. Full Western blot representative of one biological repeat, highlighting PI3K-p110α protein expression (A) and total protein expression (B) for each sample. 1, OCI-AML3 CTRL; 2, OCI-AML3 10 µM; OCI-AML3 20 µM. Table illustrates the quantified fold changes which were used to generate the OCI-AML3 PI3K-p110α expression graphs in Figure 4B, after normalising to total protein.

(A)

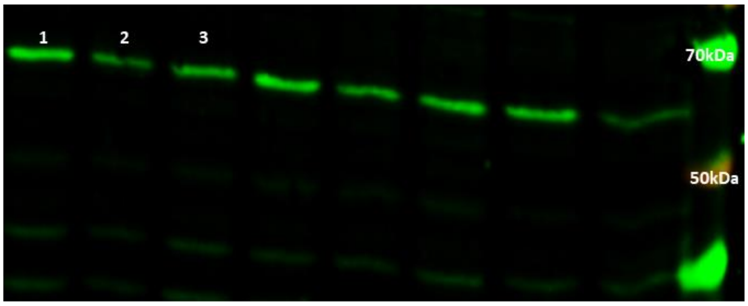


(B)

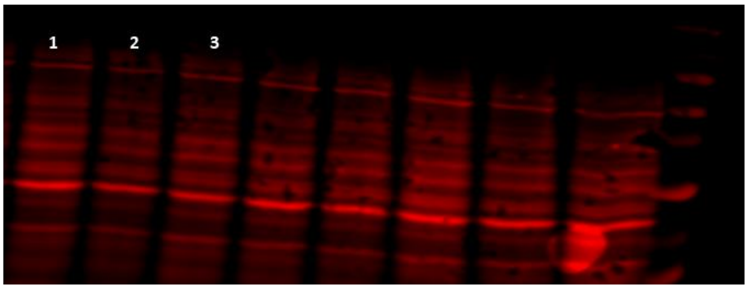


| CTRL | 10 µM | 20 µM |
|---|---|---|
| 1 | 0.493374 | 0.376781 |
| 1 | 1.235399 | 0.545981 |
| 1 | 1.142466 | 0.693069 |

**Supplementary Figure 15.** p-Akt (Ser473) expression in OCI-AML3 cells following 48 hours treatment with NUC-7738. Full Western blot representative of one biological repeat, highlighting p-Akt (Ser473) protein expression (A) and total protein expression (B) for each sample. 1, OCI-AML3 CTRL; 2, OCI-AML3 10 µM; OCI-AML3 20 µM. Table illustrates the quantified fold changes which were used to generate the OCI-AML3 p-Akt (Ser473) expression graphs in Figure 4B, after normalising to total protein.

(A)

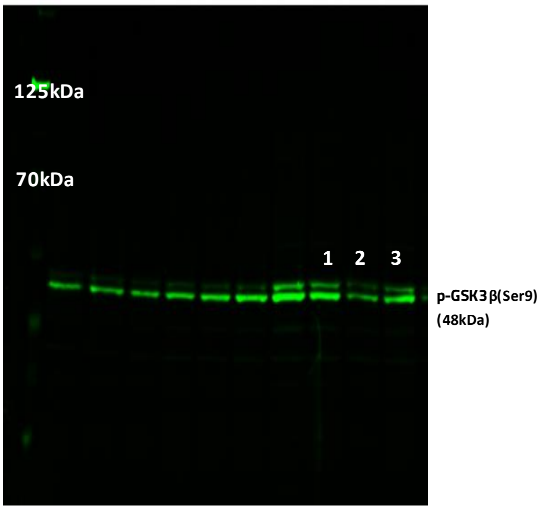


(B)

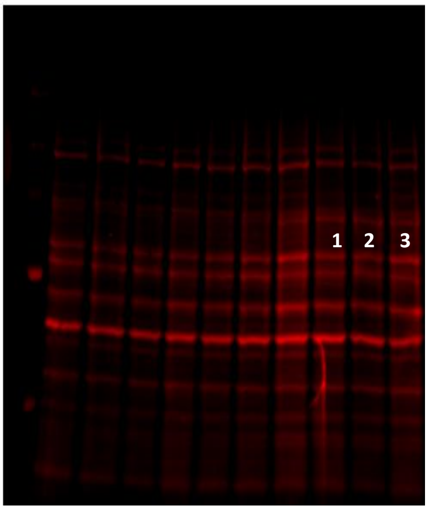


| CTRL | 10 µM | 20 µM |
|---|---|---|
| 1 | 0.94734 | 0.873181 |
| 1 | 0.859518 | 0.676811 |
| 1 | 0.955917 | 0.776031 |

**Supplementary Figure 16.** p-GSK3β(Ser9) expression in OCI-AML3 cells following 48 hours treatment with NUC-7738. Full Western blot representative of one biological repeat, highlighting p-GSK3β(Ser9) protein expression (A) and total protein expression (B) for each sample. 1, OCI-AML3 CTRL; 2, OCI-AML3 10 µM; OCI-AML3 20 µM. Table illustrates the quantified fold changes which were used to generate the OCI-AML3 p-GSK3β(Ser9) expression graphs in Figure 4B, after normalising to total protein.

(A)

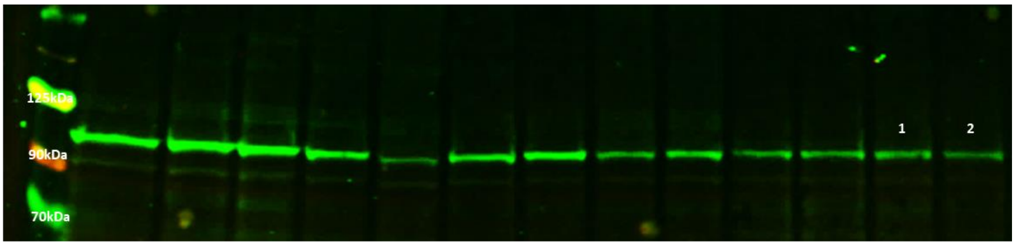


(B)

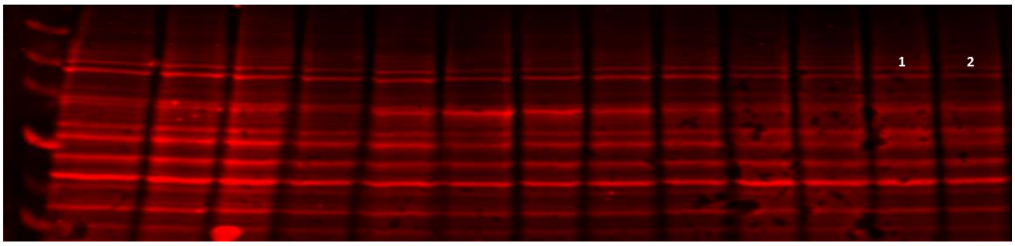


| CTRL | 10 µM |
|---|---|
| 1 | 0.39554 |
| 1 | 0.594903 |
| 1 | 0.859907 |

**Supplementary Figure 17.** PI3K-p110α expression in U937 cells following 48 hours treatment with NUC-7738. Full Western blot representative of one biological repeat, highlighting PI3K-p110α protein expression (A) and total protein expression (B) for each sample. 1, U937 CTRL; 2, U937 10 µM; U937 20 µM. Table illustrates the quantified fold changes which were used to generate the U937 PI3K-p110α expression graphs in Figure 4C, after normalising to total protein.

(A)

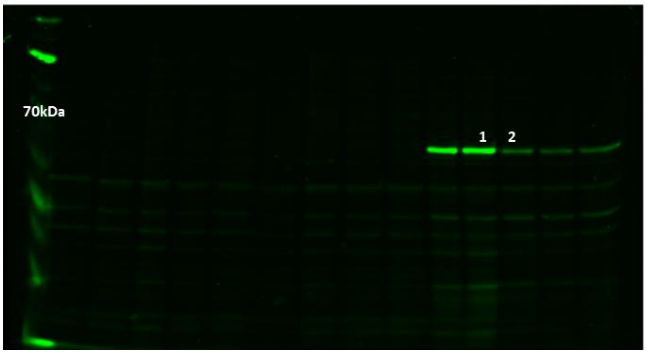


p-Akt (Ser473)
(60kDa)

(B)

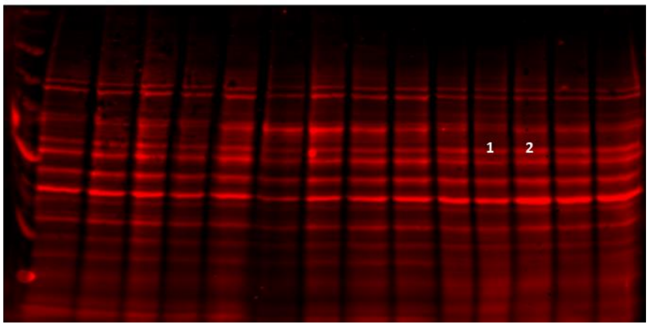


| CTRL | 10 µM |
|---|---|
| 1 | 0.167887 |
| 1 | 0.130902 |
| 1 | 0.178322 |

**Supplementary Figure 18.** p-Akt (Ser473) expression in U937 cells following 48 hours treatment with NUC-7738. Full Western blot representative of one biological repeat, highlighting p-Akt (Ser473) protein expression (A) and total protein expression (B) for each sample. 1, U937 CTRL; 2, U937 10 µM; U937 20 µM. Table illustrates the quantified fold changes which were used to generate the U937 p-Akt (Ser473) expression graphs in Figure 4C, after normalising to total protein.

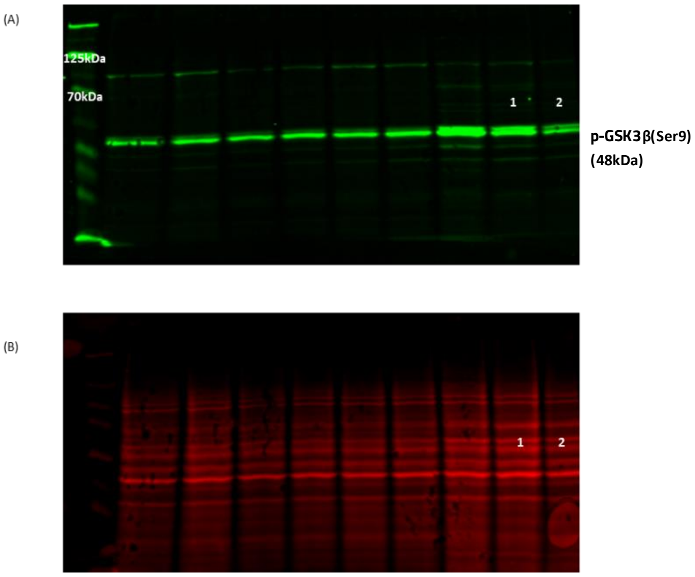


**Supplementary Figure 19.** p-GSK3β(Ser9) expression in U937 cells following 48 hours treatment with NUC-7738. Full Western blot representative of one biological repeat, highlighting p-GSK3β(Ser9) protein expression (A) and total protein expression (B) for each sample. 1, U937 CTRL; 2, U937 10 μM; U937 20 μM. Table illustrates the quantified fold changes which were used to generate the U937 p-GSK3β(Ser9) expression graphs in Figure 4C, after normalising to total protein.

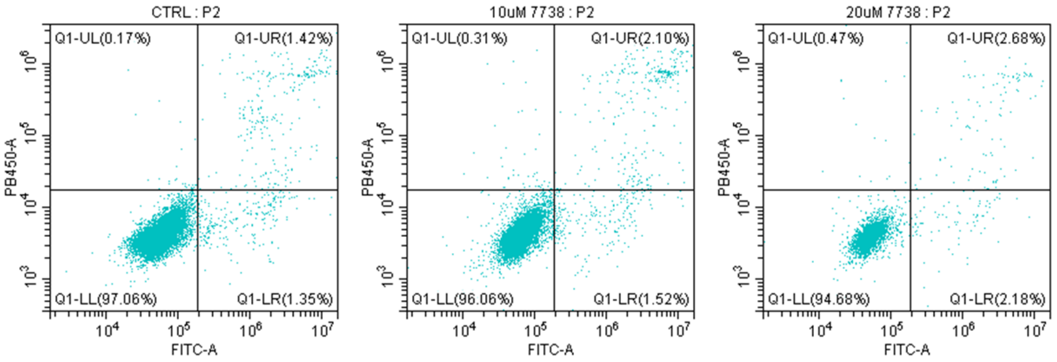


**Supplementary Figure 20.** NUC-7738 does not induce apoptosis in KG1a cells. KG1a cells were treated with NUC-7738 at 10 and 20 µM for 48 hrs and apoptosis was determined by Annexin-V DAPI staining. Dot plots generated during flow cytometry analysis highlighting the percentage of live (LL), early apoptotic (LR), late apoptotic (UR) and dead (UL) cells. (B) Gating strategy highlighted in Supplementary Figure 1. Each bar represents the mean percentage from three independent experiments with error bars indicating SD showing NUC-7738 caused a reduction in viable cells and an increase in early and late apoptotic cells in all cells examined.
